# Preliminary Note on Relief of Pain in Neuralgia

**Published:** 1913-09-15

**Authors:** George O. Jarvis

**Affiliations:** Ashland, Oregon


### PRELIMINARY NOTE ON RELIEF OF PAIN IN NEURALGIA.

BY GEORGE O. JARVIS, M.D., ASHLAND, OREGON.

Neuralgia implies a "starved" or an irritated nerve cell. If the starvation is due to deficient blood supply either in quantity or quality, then treatment is indicated which shall restore the elements in which it is deficient. An irritated nerve cell is not usually the object of direct insult, but is the recipient of a series of abnormal stimuli which arise from the peripheral pathologic *anlage*. The pain is referred to the periphery where the nerve fiber ends but the sensorium is in some nerve cell groups in the brain or cord. To relieve pain from irritation the indication is, certainly, to remove the irritant.

Needless to say, to cure a symptom one should remove the cause; but this is not always possible to do within a few hours or days in every case of neuralgia. Immediate relief from pain is an excellent thing both because it lessens suffering and because it permits all the forces of the body to direct themselves toward recovery. If the patient be worn out by lack of rest and continual pain the reparative forces of the body are less able to cope with the lesions causing the pain and unrest.

Morphin and similar drugs are not directly curative; they do not relieve pain for a long tine, and they inflict a certain amount of damage, especially if frequently repeated. Thus it is that there exists a need for a method by which one may relieve and, usually, cure neuralgic pain without the use of opium, its derivatives, or its substitutes.

Dentists and physicians commonly employ great diligence in searching for and attempting to relieve peripheral disturbing factors, but not so many sufficiently consider the fact that pain may have a central origin. If there is a central factor in trigeminal neuralgia, a tender spot will be found over the great occipital nerve just behind the mastoid process and also, occasionally, over the two upper cervical vertebrae.

No other nerve in the body has such an extensive deep origin in the brain as the trigeminus—its nuclei of origin reach from the upper part of the mesencephalon above to the upper end of the column of cells which constitute the substantia gelatinosa Rolandi of the cord. This nucleus is at the level of the second cervical vertebra.

The first posterior division of the spinal nerves is rudimentary or occasionally, altogether absent, but the second,—

the great occipital nerve—is larger than the corresponding anterior division and furnishes us a route along which we may influence the upper segments of the cord.

Dr. Albert Abrams, of San Francisco, was the first to place treatment of neuralgic affections by means directed to the central portion of the nervous system on a scientific basis. He showed that a number of supposedly surgical abdominal affections are actually neuralgic in character and that they yield to freezing applied at the appropriate level of the spine.

Neuralgia of the spinal nerves is much more amenable to this or any other treatment than neuralgia of the fifth nerve, but Dr. Abrams suggested freezing over the site of the Gasserian ganglion, just above the zygoma, and also over the two upper cervical vertebrae in trigeminal pain. He advises, in addition to the freezing, the use of the slow sinusoidal current applied with one pole over the site of the Gasserian ganglion and the other over the upper cervical vertebrae. As everyone does not possess a sinusoidal apparatus freezing is of more general service.

Freezing is done as follows: Ether is sprayed on the part to be frozen, from an atomizer, operated either by hand or by compressed air. Trial has shown that Malinkrodt's ether is perhaps the best made for this purpose. If the air contains much moisture it may be necessary to start the freezing with a little ethyle chlorid sprayed on before the ether is begun. Dr. W. T. Baird, of El Paso, Texas, employs a chunk of ice, dipped in salt and pressed against the point of vertebral tenderness for about three minutes. The depression left may then be frozen with ether spray. Dr. Guild, of Des Moines, Iowa, says that this method is superior to that with ether spray as it may be maintained for a longer time and with less sloughing or desquamation.

Freezing should be done thoroughly and continued for two or more minutes, and repeated at intervals of three or four days till the desired effect is produced. The frozen skin will become hyperemic and a little sore, so that it may be necessary to wait some days between treatments. Painting the skin after freezing with collodion mitigates somewhat this skin irritation and, by freezing over the collodion at the next sitting, a second treatment may be applied sooner than would otherwise be the case.

The great occipital on the affected side is the more tender and treatment will be first directed to that point; but from the fact that both great occipital nerves are connected with the same cord segments, freezing on either side will produce a similar—though not as marked—effect. This point may be remembered with advantage if one desires to apply the methol frequently and yet fears the skin irritation produced by rapidly repeated freezings.

In freezing over the site of the Gasserian ganglion, which is a decidedly more painful procedure than over the spinal centers, one cannot make use of either side at will, but is compelled to spray over the affected side.—*Pacific Gazette.*

